# MitoQ Modulates Lipopolysaccharide-Induced Intestinal Barrier Dysfunction via Regulating Nrf2 Signaling

**DOI:** 10.1155/2020/3276148

**Published:** 2020-04-11

**Authors:** Shengfeng Zhang, Qingniao Zhou, Youcheng Li, Yunli Zhang, Yinmei Wu

**Affiliations:** ^1^Department of Intensive Care Medicine, The People's Hospital of Guangxi Zhuang Autonomous Region, China; ^2^School of Preclinical Medicine, Guangxi Medical University, China; ^3^Guigang City People's Hospital, China; ^4^The Guangxi Zhuang Autonomous Region Work's Hospital, China

## Abstract

**Background:**

Gut barrier dysfunction with alterant mucosal permeability during sepsis is a challenge problem in clinical practice. Intestinal epithelial cells (IECs) are strongly involved in mucosal oxidative stress and inflammatory response. The current study aimed at investigating the effect of MitoQ, a mitochondrial targeted antioxidant, in the treatment of intestinal injury and its potential mechanism during sepsis.

**Methods:**

30 minutes before sepsis induction by lipopolysaccharide (LPS) treatment, mice were treated with MitoQ. Intestinal histopathology, mucosal permeability, inflammatory cytokines, and mucosal barrier proteins were evaluated in the present study.

**Results:**

MitoQ pretreatment significantly decreased the levels of plasma diamine oxidase, D-lactate, and intestinal histological damage and markedly restored the levels of tight junction proteins (ZO-1 and occludin) following LPS challenge. Furthermore, MitoQ inhibited the LPS-induced intestinal oxidative stress and inflammatory response, evidenced by increased levels of intestinal superoxide dismutase and glutathione, and decreased levels of intestinal IL-1, IL-6, TNF-*α*, and nitric oxide levels. Mechanically, we found that MitoQ inhibited the oxidative stress via activating nuclear factor E2-related factor 2 (Nrf2) signaling pathway and its downstream antioxidant genes, including HO-1, NQO-1, and GCLM.

**Conclusions:**

MitoQ exerts antioxidative and anti-inflammatory effects against sepsis-associated gut barrier injury by promoting Nrf2 signaling pathway.

## 1. Introduction

Sepsis is a challenge problem that usually induces multiple organ injuries and is the major cause of death in the critical care units [1]. The gut has been indicated as the “central organ” of multiple organ failure (MOF) in sepsis. The dysfunction of gut barrier provides a route for enteric pathogenic organisms from gut to the mesenteric lymph and the circulation, inducing systemic inflammatory response, MOF, and septic shock [2]. The intestinal function can be affected by a variety of factors, including inflammatory response, bacterial challenge, and oxidative stress [3]. Therefore, repair and maintenance of gut barrier serve as a potential target in treating sepsis.

Increased evidence indicated that mitochondrial oxidative injury could disrupt mitochondrial integrity and inhibit the production of mitochondrial ATP, which play a critical role in inducing cell death and MOF during sepsis [4]. Consistent with an important role for mitochondrial function in sepsis, previous study reported that level of oxidative stress was significantly increased in sepsis, shown by increased levels of radicals and lipid peroxides, and decreased antioxidant capacity [5]. Additionally, mitochondrial oxidative damage was demonstrated in animal models of sepsis. There is now growing evidence that mitochondrial oxidative injury and oxidative stress serve as central pathological mechanisms in sepsis-induced organ injuries [5]. Increased oxidative stress could activate various signaling pathways, and excessive reactive oxygen species (ROS) can induce the death of intestinal epithelial cells, amplify inflammation, and damage gut barrier during the progress of sepsis [6].

Mitochondrial dysfunction induced by oxidative stress play an important role in the development of sepsis, leading to the impaired intestinal injury. A recent study demonstrated a strengthful effect of the mitochondrially targeted antioxidant MitoQ on alleviating oxidative injury and improving mitochondrial function [7]. However, no studies have provided strong evidence on the beneficial role of MitoQ in sepsis-induced gut injury. MitoQ as a mitochondrially targeted antioxidant comprises a triphenyl phosphonium and coenzyme Q10, enabling its latent ability of being accumulated within the mitochondria [8]. Because of MitoQ's strong antioxidant ability, MitoQ has been suggested to play a protective role in a variety of diseases, including ischemia reperfusion (IR) and liver fibrosis [9, 10]. Particularly, Hu et al. [11] recently indicated that MitoQ could alleviate the intestinal injury via activating nuclear factor E2-related factor 2 (Nrf2) signaling following IR. Therefore, we hypothesized that MitoQ can protect against the sepsis-induced intestinal injury.

In the present study, we investigated the potential effect of MitoQ on gut barrier function during sepsis. Our results suggested that pretreatment of MitoQ prevented the intestinal mucosal injury, intestinal hyperpermeability, and bacterial translocation and also modulates intestinal inflammatory response during sepsis. Mechanically, we showed that MitoQ improved oxidative stress though activating Nrf2 signaling pathway.

## 2. Methods

### 2.1. Animals

Male C57BL/6 mice (aged from 8 to 10 weeks) obtained from the Model Animals Research Center of Nanjing University were maintained under specific conditions in a temperature-controlled room. The animal study is designed and performed in accordance with the principles of the Declaration of Helsinki and with approval from the institutional animal ethical committee of The People's Hospital of Guangxi Zhuang Autonomous Region.

### 2.2. Animal Model of Sepsis

After more than 7 days of acclimation, the mice were randomly allocated into four groups: (a) control group, in which the mice were injected with saline (10 mg/kg, ip); (b) MitoQ group, in which mice were administrated with MitoQ prior to saline; (c) LPS group, in which mice were given LPS (10 mg/kg dissolved in saline, ip); (d) LPS+ MitoQ group, in which mice were treated with MitoQ prior to LPS injection. MitoQ (4 mg/kg; added as MitoQ adsorbed to *β*-cyclodextran) in100 *μ*l 0.9% saline was injected into the tail vein 15 min before the onset of sepsis induction. The following symptoms in mice were observed to judge the success of the sepsis model: mouse lethargy, reduced activity, slow movements, erected back hair, sticky discharge from the eyelids, anal stool adhesions, and turbid urine.

IEC-6, the intestinal epithelial cell line, was purchased from the American Type Culture Collection (Rockville, MD, USA). IEC-6 cells were treated with 1.0 *μ*M MitoQ for 6 h prior to LPS treatment. DMSO was used as a control.

### 2.3. Histopathological Assessment of Intestines

Twenty-four hours after LPS or saline intraperitoneal injection, about 1 cm ileal segments were fixed in paraformaldehyde and then embedded in paraffin. 4 *μ*m of paraffin sections were stained by hematoxylin and eosin (H&E) for light microscopy. Histological score of intestine injury was assessed according to the instructions as previously described [11]. Histological score for every section was evaluated blindly.

### 2.4. Measurement of Intestinal Permeability and Bacterial Translocation

The mice were fasted for 4 hours and then given fluorescein isothiocyanate (FITC)-dextran (FD-40; 4 kDa; Sigma) by gavage (600 mg kg^−1^). The mice were sacrificed and bled by cardiac puncture after 4 hours. Serum FITC concentration was detected by fluorometry.

Mesenteric lymph nodes (MLN) was taken using aseptic techniques. Each node (0.1 g) was homogenized in a tissue grinder after collecting tissue samples. 100 *μ*l diluted homogenates were cultured on Mac-Conkey's agar for 24 hours at 37°C. Bacterial growth on the plates was quantified as colony-forming units/g of tissue.

### 2.5. Immunofluorescence Assessment

The immunofluorescence analysis was used to evaluate the location and expression of occluding in intestinal tissues, and the IEC-6 cells grown in 24-well cell culture plates was used to analyse the colocalization of cell nucleus and Nrf2. The intestinal tissues and the IEC-6 cells were washed with phosphate-buffered saline (PBS) and fixed with 4% paraformaldehyde (*w*/*v*) for 20 min at room temperature. After washing three times with PBS for 5 min, the fixed coverslips were permeabilized in PBS with 0.1% Triton X-100 for 5 min at room temperature. Nonspecific sites were blocked within PBS for 1 h at 37°C. The 1 : 100 dilutions of rabbit polyclonal antibodies against occluding (Abcam, Cambridge, UK) and Nrf2 (Santa Cruz Biotechnology, Dallas, TX, USA) were incubated at 4°C overnight according to the manufacturer's instructions. After washing with BSA/PBS (three times), the sections were probed with their respective FITC-conjugated secondary IgG antibodies. Slides incubated in the absence of primary antibodies were used as negative controls. Finally, images were captured by fluorescence confocal microscopy (Leica Microsystems, Heidelberg GmbH, Mannheim, Germany).

### 2.6. RNA Isolation and Quantitative Real-Time PCR (qRT-PCR)

Total RNA was extracted from intestinal tissues and IEC-6 cells using Trizol reagent (Invitrogen, USA), according to the manufacturer's instructions. First-strand cDNAs were synthesized from 1 *μ*g with a Prime Script RT reagent kit (Takara). The qRT-PCR was performed using a SYBR green PCR kit and a MyiQ Single Color Real-time PCR Detection System (Bio-Rad Laboratories, USA). The cDNA amplifications were performed with the primers in Supplementary Table 1. The PCR conditions were as follows: 95°C for 15 min, followed by 40 cycles of 95°C for 15 s and 60°C for 30s. Reactions were run in duplicate using RNA samples from three independent experiments. The fold change in expression of each gene was calculated using the 2-*Δ*Ct (*Δ*Ct, relative cycle threshold compared with GAPDH) method.

### 2.7. Biochemical Assay

The indicators of oxidative stress were evaluated using malondialdehyde (MDA), superoxide dismutase (SOD), and glutathione (GSH) (Nanjing Jiangcheng, China), as previously described [12]. Proinflammatory cytokines in serum and intestines, including tumor necrosis factor (TNF-*α*), IL-1*β*, and IL-6, were measured by using ELISA kits based on the manufacture's instruction. Plasma and intestinal nitric oxide (NO) levels were detected by using Griess Reagent kit, as previously described. The concentration of diamine oxidase (DAO), D-lactate (D-lac), and lactic dehydrogenase (LDH) were assessed through commercial kits according to the manufacturer's recommendation.

### 2.8. Statistical Analysis

The results were expressed as means ± standard deviation (SD) and were analyzed by SPSS 17.0 (Chicago, IL, USA) and Graphpad Prism software 6.0 (La Jolla, CA, USA). Student's *t*-test or one-way analysis of variance was performed to compared the continuous variables between groups. Differences in survival rates between groups were used the log-rank tests. All *p* values < 0.05 was considered significant.

## 3. Results

### 3.1. MitoQ Ameliorates LPS-Induced Intestinal Injury

**Figure 1:**
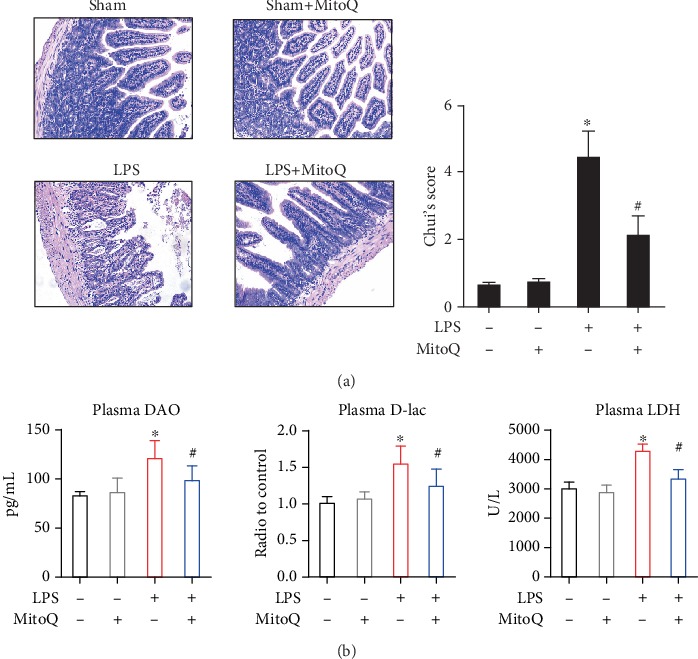
MitoQ ameliorates LPS-induced intestinal injury. (a) Representative images of intestinal histology (H&E staining); (b) Levels of DAO, D-lac, LDH in plasma. Data are expressed as the mean ± SD. ^∗^*P* < 0.05 vs. control group; ^#^*P* < 0.05 vs. LPS group.

As shown in Figure 1, H&E staining demonstrated that LPS-treated mice exhibited damaged intestinal villi, inflammatory cell filtration, and local cell death, while pretreatment with MitoQ preserved the integrity of intestinal structures and alleviated the inflammatory infiltration compared with the LPS stimulation (Figure 1(a)). Additionally, LPS stimulation induced a significant increase in plasma D-lac, DAO, and LDH, while MitoQ pretreatment reduced the up-regulation of these tissue injury biomarkers following LPS injection (Figure 1(b)).

### 3.2. MitoQ Improved Intestinal Permeability and Inhibited Bacterial Translocation during LPS-Induce Sepsis

**Figure 2:**
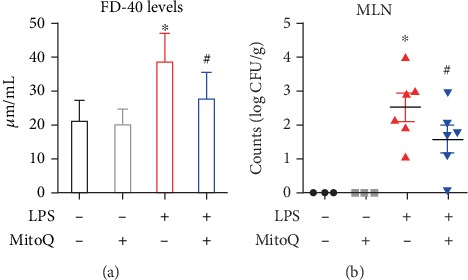
MitoQ improved intestinal permeability and inhibited bacterial translocation during LPS-induce sepsis. (a) Serum FD-40 level was evaluated in vivo permeability. (b) Bacterial CFU was quantified in MLN, and each data point represents the CFU from each mouse. CFU: bacterial colony-forming units; MLN: mesenteric lymph nodes. Data are expressed as the mean ± SD. ^∗^*P* < 0.05 vs. control group; ^#^*P* < 0.05 vs. LPS group.

The results related to gut permeability were consistent with our histological findings. FD4, the paracellular flux of a fluorescent marker, was measured to evaluate the intestinal permeability [13]. Our sepsis mice showed an increase in the intestinal permeability compared with the control group. Concomitantly, pretreatment of MitoQ reduced the epithelial permeability (Figure 2(a)). The injury of the gut barrier initiates the passage of bacteria from gut to mesenteric lymph (MLN). Expectedly, LPS treatment contributed to significant bacterial translocation to the MLN. However, the mice pretreated with MitoQ decreased the counting number of bacteria in the MLN (Figure 2(b)).

### 3.3. Effects of MitoQ on Intestinal Tight Junctions during Sepsis

**Figure 3:**
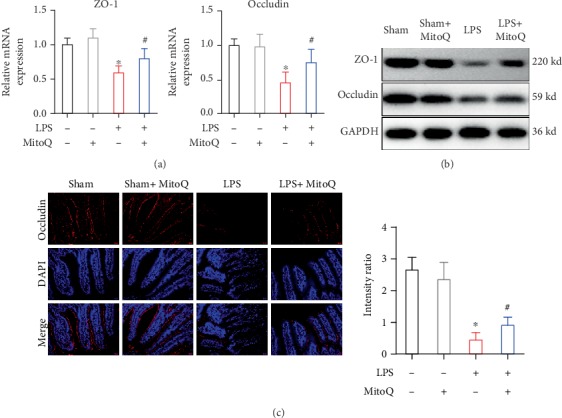
Effects of MitoQ on intestinal tight junctions during sepsis. (a) mRNA and (b) protein levels of ZO-1 and occludin were evaluated by qPCR. (c) Expression and location of tight junction protein (occludin) in the intestinal mucosa by immunofluorescence. ^∗^*P* < 0.05 vs. control group; ^#^*P* < 0.05 vs. LPS group.

Tight junction (TJ) protein serves as an important role in maintaining intestinal barrier function. Compared with the control group, LPS stimulation suppressed intestinal occludin and ZO-1 via qPCR analysis and Western blot. Pretreatment with MitoQ increased the mRNA and protein expressions of occludin and ZO-1 (Figures 3(a) and 3(b)). Additionally, immunofluorescence of occludin was used to assess TJ protein of the gut. Staining of occludin showed a lack of focus staining within the surfaces of epithelial cells and some villi of the sepsis-injured mice, whereas MitoQ markedly alleviated these effects (Figure 3(c)).

### 3.4. The Effects of MitoQ on Intestinal Oxidative Stress in Sepsis

**Figure 4:**
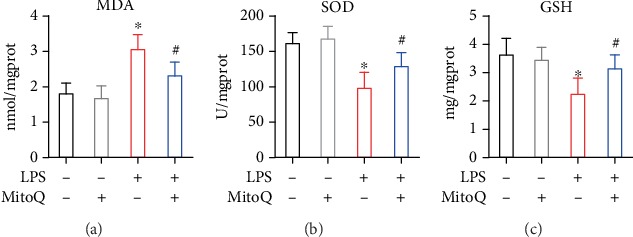
The effects of MitoQ on intestinal oxidative stress in sepsis. The effects of MitoQ on MDA, SOD, and GSH in the intestinal mucosa following LPS infection. Data are expressed as the mean ± SD. ^∗^*P* < 0.05 vs. control group; ^#^P < 0.05 vs. LPS group.

LPS stimulation induced the production of MDA in the intestines compared with the control group, while supplementation with MitoQ significantly decreased MDA level (Figure 4(a)). Both of the intestinal SOD and GSH levels were measured to assess the enzymatic activities, and the levels of these two antioxidase activities were significantly decreased with LPS challenge. However, MitoQ pretreatment enhanced their enzymatic activities (Figures 4(b) and 4(c)).

### 3.5. MitoQ Decreases Intestinal and Systemic Inflammatory Agents in Sepsis

**Figure 5:**
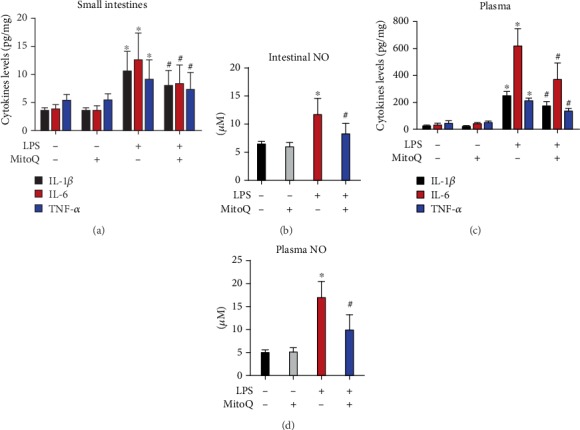
MitoQ decreases intestinal and systemic inflammatory agents in sepsis. Inflammatory cytokines in plasma (a) and intestines (b) were measured by ELISA kits. Plasma and intestinal NO levels were evaluated by Griess Reagent kit. Data are expressed as the mean ± SD. ^∗^*P* < 0.05 vs. control group; ^#^*P* < 0.05 vs. LPS group.

Systemic and intestinal proinflammatory cytokines were detected to evaluate the effect of MitoQ in inflammatory response. The levels of TNF-*α*, IL-1, and IL-6 in intestines and plasma were increased following LPS challenge compared with control mice (Figures 5(a) and 5(c)). Mice pretreated with MitoQ led to marked reduction in these inflammatory cytokines at the same time period. Furthermore, intestinal and systemic NO levels were decreased in mice pretreated with MitoQ prior to LPS stimulation (Figures 5(b) and 5(c)).

### 3.6. MitoQ Alleviates LPS-Induced Oxidative Stress via Nrf2 Signaling

**Figure 6:**
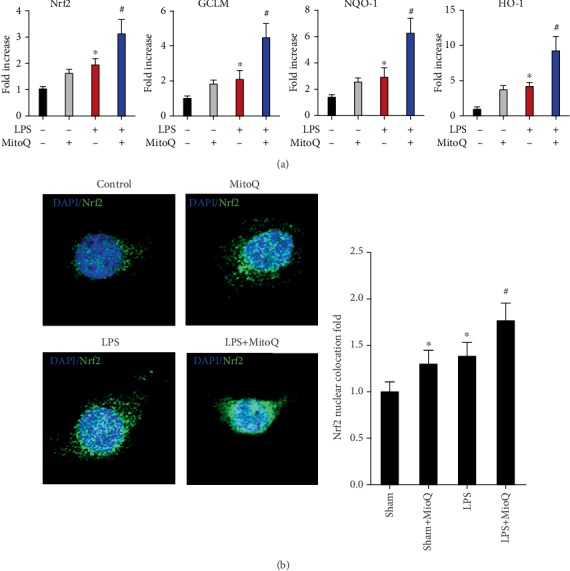
MitoQ alleviates LPS-induced oxidative stress via Nrf2 signaling. (a) qPCR analysis of total Nrf2, GCLM, NQO-1, and HO-1 among four groups. Immunofluorescence staining showing changes in Nrf2 fluorescence (b). MitoQ increases nuclear translocation of Nrf2 (endogenous) in LPS-treated IEC-6 cells.

The effects of MitoQ on sepsis-mediated Nrf2, GCLM, NQO-1, and HO-1 levels were measured via real-time PCR assay. As shown in Figure 6(a), Nrf2-related antioxidant genes were upregulated following LPS challenge or MitoQ group compared with control mice. The MitoQ pretreatment following LPS stimulation showed increased the most in the expression of these antioxidant genes. To investigate the effect of MitoQ on the nuclear translocation of Nrf2, nuclear import of Nrf2 in IEC-6 cells was performed using immunofluorescence. Our results suggested that nuclear Nrf2 within IEC-6 cells was significantly increased in the MitoQ+ LPS group compared with control and LPS group (Figure 6(b)).

## 4. Discussion

Sepsis is a severe challenge problem with a high rate of mortality, despite the improved management of septic patients, including fluid resuscitation, antibiotic therapy, and advance surgical approaches [14]. The gut has been defined as the driver of MOF during sepsis [15]. The impairments of gut during sepsis are mainly reflected in the disruption of epithelial integrity, leading to the intestinal hyperpermeability. Increased gut permeability was directly associated with bacterial translocation, systemic inflammatory response, and MOF [16]. In the present study, we showed that pretreatment of MitoQ alleviated the LPS-induced intestinal mucosal injury. Phenotypically, MitoQ pretreatment increased the expression of tight junction proteins, ameliorated oxidative stress, and reduced intestinal and systemic inflammatory response. Additionally, we observed that protective effect of MitoQ appeared to be accomplished by upregulating antioxidants genes, including Nrf2, HO-1, NQO-1, and GCLM. These results indicated that MitoQ protects against sepsis-induced intestinal barrier injury via activating Nrf2 signaling pathway.

MitoQ, as a powerful activator of the Nrf2 pathway, plays a critical role in cytoprotective and antiapoptotic effect in various diseases, and these protective properties were associated with the decrease of inflammatory response and oxidative stress [7]. Our study demonstrated that MitoQ pretreatment can attenuate the intestinal injury induced by LPS. Our results showed that LPS injection induced the injury of intestinal villi and increased the biomarkers of intestinal injury (DAO, and D-lac). However, these changes were markedly blocked by pretreatment of MitoQ, which suggested that MitoQ improved intestinal barrier function.

Tight junctions are important factors in regulating intestinal barrier function and maintaining epithelial permeability [17]. A series of tight junction proteins, such as ZO-1 and occludin, contribute to the formation and shaping of intercellular tight junction. In our study, we investigated that the expressions of ZO-1 and occludin, the crucial components of tight junction, were obviously decreased in the intestine of LPS-treated mice. However, MitoQ prevented LPS-induced down-regulation of ZO-1 and occludin. Our data indicated that MitoQ could protect intestinal integrity following LPS challenge through regulating intracellular tight junction.

Increased oxidative stress is associated with increased mortality during sepsis. Previous studies showed that LPS exposure could cause oxidative stress in the gut, and antioxidant administration was suggested as a potential therapy for protecting against organ injuries in experimental studies [18, 19]. However, treatment with antioxidant in sepsis patients could not be effective. Increased evidence suggested that mitochondrial protection against oxidative stress may be particularly critical and effective in critically ill patients [20]. Hu et al. [11] recently demonstrated that mitochondrial integrity was damaged in critical ill patients, and they further showed that mitochondria-targeted antioxidants significantly protected against ischemia-/reperfusion-associated organ injuries. They also indicated that MitoQ could inhibit the activities of respiratory chain complexes I, III, and IV and restored the mitochondrial ATP production. In our study, we used a mitochondria-targeted antioxidant MitoQ that has been indicated to accumulate in all organs. Our results showed that MitoQ markedly reduced the level of MDA as well as a rise of SOD and GSH. Therefore, our results indicated that MitoQ could inhibit the oxidative stress during intestinal injury in sepsis.

Sepsis is a systemic activation of the innate immune response because of pathogen infections, involving increased inflammatory response induced by cellular oxidative insults [21]. The unbalance of inflammatory homeostasis causes cellular disruption, organ injury, and even death. Uncontrolled intestinal inflammation is an important factor to lead to the epithelial damage and gut permeability in sepsis [22]. IL-1 and TNF-*α* were suggested to directly cause intestinal mucosal injury, and IL-6 was proved to play a critical role in the persistence of inflammatory response. Previous study also confirmed a direct correlation between increased inflammation and the disruption of intestinal barrier integrity [22]. Additionally, increased production of proinflammatory cytokines causes the loss of tight junction proteins. Therefore, inhibition of these proinflammatory cytokines should be an effective therapy to alleviate intestinal inflammatory injury. In our study, we demonstrated that MitoQ pretreatment markedly decreased the levels of intestinal and systemic proinflammatory mediators, including TNF-*α*, IL-1*β*, and IL-6, which indicated that MitoQ can suppress inflammatory responses.

Nitric oxide was suggested to regulate inflammatory response during sepsis [23]. Enhanced production of nitric oxide is associated with the intestinal barrier injury during inflammation [24]. The inhibition of nitric oxide secretion significantly protect against tissue injuries during sepsis [23]. In the present study, pretreatment of MitoQ can inhibit the production of intestinal and systemic nitric oxide following LPS stimulation, which further confirms potential anti-inflammatory properties of MitoQ.

Nrf2 signaling plays an irreplaceable factor in oxidative stress-induced tissue injures and activation of Nrf2 pathway protected cells from oxidative insults [11, 25]. Upon the activation of oxidative insults, Nrf2 translocates from cytoplasm to nucleus and subsequently activates downstream antioxidant genes [26]. Additionally, increased evidence suggested that Nrf2 signaling pathway is an important modulator in anti-inflammatory responses. The activation of Nrf2 signaling was showed to prevent inflammation and inflammatory cell infiltration and reduce organ injuries [27]. Therefore, we investigate whether MitoQ can protect against LPS-induced oxidative insults through activating Nrf2 signaling pathway. Our results showed that MitoQ could increase the expression of the Nrf2-releated antioxidant genes and induce the nuclear translocation of Nrf2 in IEC-6 following LPS stimulation. These data suggest a crucial role of MitoQ on the activation of Nrf2 signaling pathway during sepsis, but the precise mechanisms remains to be further clarified.

MitoQ is suggested to involve in different animal and cell models due to its protective effect in various types of oxidative injury. In sepsis, injury of intestinal epithelium contributes to a damaged gut barrier function and the bacterial translocation. In our study, pretreatment of MitoQ can alleviate intestinal barrier dysfunction following LPS challenge. Mechanically, the protective properties of MitoQ may be associated with the improvement of antioxidant and anti-inflammatory function via activating Nrf2 signaling pathway. Therefore, MitoQ has the potential to be an effective therapy in sepsis.

## Data Availability

The data used to support the findings of this study are included within the article.

## Abbreviations

IECs: Intestinal epithelial cells
LPS: Lipopolysaccharide
Nrf2: Nuclear factor E2-related factor 2
MOF: Multiple organ failure
ROS: Reactive oxygen species
PBS: Phosphate buffered saline
qRT-PCR: Quantitative real-time PCR
MDA: Malondialdehyde
SOD: Superoxide dismutase
GSH: Glutathione
TNF-*α*: Tumor necrosis factor
NO: Nitric oxide
DAO: Diamine oxidase
D-lac: D-lactate
LDH: Lactic dehydrogenase
SD: Standard deviation
MLN: Mesenteric lymph
TJs: Tight junctions.

## Supplementary Materials

Supplementary Materials Supplementary table 1: primers used for quantitative PCR analysis.
