# Supplementary material for: MitoQ Modulates Lipopolysaccharide-Induced Intestinal Barrier Dysfunction via Regulating Nrf2 Signaling
Source: Mediators Inflamm. 2020 Apr 11;2020:3276148. doi: 10.1155/2020/3276148 (PMC7171662; doi:10.1155/2020/3276148)
Supplement: Supplementary Materials — Supplementary table 1: primers used for quantitative PCR analysis. [file 3276148.f1.doc]

Supplementary Table 1. Primers used for quantitative PCR analysis

| **Genes** | **Primers sequence** |
| --- | --- |
| Nrf-2 | F: 5’- GGACATGGAGCAAGTTTGGC -3’  R: 5’- GGGCTGGGGACAGTGGTAGT -3’ |
| GCLM | F: 5’- ATCATGGCTTCCCCTCCAAT -3’  R: 5’- CCTCCCAGTAAGGCTGCAAAT -3’ |
| NQO-1 | F: 5’- CGGTGAGAAGAGCCCTGAT -3’  R: 5’- CGACCACCTCCCATCCTT -3’ |
| HO-1 | F: 5’- TCACCTTCCCGAGCATCGA -3’  R: 5’- GGCGGTCTTAGCCTCTTCTGT -3’ |
| ZO-1 | F: 5’- GCCGCTAAGAGCACAGCAA -3’  R: 5’- GCCCTCCTTTTAACACATCAGA -3’ |
| Occludin | F: 5’- TGAAAGTCCACCTCCTTACAGA -3’  R: 5’- CCGGATAAAAAGAGTACGCTGG -3’ |
| GAPDH | F: 5’- GGCACAGTCAAGGCTGAGAATG -3’  R: 5’- ATGGTGGTGAAGACGCCAGTA -3’ |
